# Protein kinase RNA- like endoplasmic reticulum kinase (PERK) signaling pathway plays a major role in reactive oxygen species (ROS)- mediated endoplasmic reticulum stress- induced apoptosis in diabetic cardiomyopathy

**DOI:** 10.1186/1475-2840-12-158

**Published:** 2013-11-02

**Authors:** Zhong-Wei Liu, Hai-Tao Zhu, Kun-Lun Chen, Xin Dong, Jin Wei, Chuan Qiu, Jia-Hong Xue

**Affiliations:** 1 Department of Cardiology, Second Affiliated Hospital, Xi’an Jiaotong University, Xi’an, Shaanxi, China; 2 School of Medicine, Xi’an Jiaotong University, Xi’an, Shaanxi, China; 3 Department of Biostatistics & Bioinformatics, School of Public Health & Tropical Medicine, Tulane University, New Orleans, LA, USA

**Keywords:** Diabetic cardiomyopathy, Apoptosis, Oxidative stress, Endoplasmic reticulum stress

## Abstract

**Background:**

Endoplasmic reticulum (ER) stress is considered one of the mechanisms contributing to reactive oxygen species (ROS)- mediated cell apoptosis. In diabetic cardiomyopathy (DCM), cell apoptosis is generally accepted as the etiological factor and closely related to cardiac ROS generation. ER stress is proposed the link between ROS and cell apoptosis; however, the signaling pathways and their roles in participating ER stress- induced apoptosis in DCM are still unclear.

**Methods:**

In this study, we investigated the signaling transductions in ROS- dependent ER stress- induced cardiomocyte apoptosis in animal model of DCM. Moreover, in order to clarify the roles of IRE1 (inositol - requiring enzyme-1), PERK (protein kinase RNA (PKR)- like ER kinase) and ATF6 (activating transcription factor-6) in conducting apoptotic signal in ROS- dependent ER stress- induced cardiomocyte apoptosis, we further investigated apoptosis in high- glucose incubated cardiomyocytes with IRE1, ATF6 and PERK- knocked down respectively.

**Results:**

we demonstrated that the ER stress sensors, referred as PERK, IRE1 and ATF6, were activated in ROS- mediated ER stress- induced cell apoptosis in rat model of DCM which was characterized by cardiac pump and electrical dysfunctions. The deletion of PERK in myocytes exhibited stronger protective effect against apoptosis induced by high- glucose incubation than deletion of ATF6 or IRE in the same myocytes. By subcellular fractionation, rather than ATF6 and IRE1, in primary cardiomyocytes, PERK was found a component of MAMs (mitochondria-associated endoplasmic reticulum membranes) which was the functional and physical contact site between ER and mitochondria.

**Conclusions:**

ROS- stimulated activation of PERK signaling pathway takes the major responsibility rather than IRE1 or ATF6 signaling pathways in ROS- medicated ER stress- induced myocyte apoptosis in DCM.

## Background

The rapidly growing morbidity and mortality of diabetes mellitus (DM) makes it a prevailing disease globally [1]. As an independent risk factor, hyperglycemia in DM is responsible for various cardiovascular complications. Characterized by consistent diastolic dysfunction and ventricular hypertrophy, diabetic cardiomyopathy (DCM) often develops in diabetic patients [2]. In diabetic population, risk and morbidity of developing congestive heart failure increased significantly [3,4]. Apoptosis of cardiomyocytes is considered as a key pathological change in DCM [5]. Studies have confirmed that cardiomyocyte apoptosis is the cause of contractile units lost and reparative fibrosis in DCM [6]. It is believed that cardiomyocyte apoptosis increased in hearts from streptozotocin (STZ)- induced diabetic animals [7].

Excessive production of reactive oxygen species (ROS) is found in diabetic hearts from both type 1 and type 2 diabetes [8,9]. Hyperglycaemia- induced ROS generation is considered to be responsible for progression and development of DCM [10], because ROS not only induces oxidative stress, but also impairs antioxidant defense system in cardiomyocytes [11]. Some cell death leading maladaptive signaling pathways are activated by excessive ROS production, contributing to pathogenesis of DCM [12]. Mitochondria is believed the major source of ROS under condition of hyperglycaemia because excessive ROS is generated by mitochondrial glucose oxidation [13].

As an important specialized organelle, endoplasmic reticulum (ER) is involved in protein folding, protein maturation, lipid synthesis, and calcium homeostasis [14]. Malfunctions of ER induced by various factors could lead to unfolded proteins response (UPR), resulting in ER stress [15]. Previous study provided the evidence for the involvement of the ER stress in the cardiac apoptosis in diabetic rat model [7]. These experimental data suggested that ER stress was initiated in diabetic hearts, and the ER stress- induced apoptosis took part in the pathogenesis and development of DCM. Investigations have also demonstrated that ER stress induces cell apoptosis independently from mitochondria- and death receptor- dependent pathways [16].

Concurrently, ER stress is often accompanied by increased ROS generation [17]. Excessive ROS which initiates the perturbation of cellular redox balance causes cell apoptosis [18]. It occurs that ROS is one of the important stimuli that trigger ER stress [19], a paradigm called ROS- dependent ER stress. However, the molecular events linking ROS and the components of the ER stress are still unclear. In mammalian cells, UPR signaling is conducted by key three major ER resident transducers- governed signaling pathways to induce apoptosis under severe ER stress, namely the inositol- requiring enzyme-1 (IRE1), activating transcription factor-6 (ATF6) and protein kinase RNA (PKR)- like ER kinase (PERK) [20,21]. Given the critical role of IRE1, PERK and ATF6 in UPR signaling, it is most likely that these receptors would contribute a lot to ER stress- induced apoptosis. Thus, clarifying the changes in these pathways would be very helpful in learning the mechanism of cardiac apoptosis in response to ROS which is induced by hyperglycaemia in DCM.

In this study, we investigated the signaling transductions in ROS- dependent ER stress- induced cardiomocyte apoptosis in animal model of DCM. Moreover, in order to clarify the roles of IRE1, PERK and ATF6 in conducting apoptotic signal in ROS- dependent ER stress- induced cardiomocyte apoptosis, we further investigated apoptosis in high- glucose incubated cardiomyocytes with IRE1, ATF6 and PERK- knocked down respectively. N- acetylcysteine (NAC), the ROS scavenger, was also used to strengthen the findings concerning relationships between ROS, ER- stress and apoptosis in this study. Results in this study would be helpful in understanding the mechanism(s) of DCM and thus proposing potential therapeutic target(s) in treatment of DCM.

## Methods

### Animals

40 female Sprague- Dawley rats from Animal Experimental Center of Xi’an Jiaotong University (SPF class, 9 weeks old, mean body weight 291 ± 15 g) were used in this study. Rats were raised in 4 independent polypropylene cages under controlled conditions (artificial alternating 12-hour light- dark cycle; humidity 65% ± 4%; temperate 25 ± 1°C) for 1 week before implementation of experiments. Rats were allowed to access freely to standard rat diet and fresh tap water continuously. All animal experimental procedures were carried out in accordance with protocols approved by Medical Animal Research Ethics Committee at Xi’an Jiaotong University.

### Animal grouping and treatment

40 rats were randomly assigned into 4 groups: control group (Ctrl, n = 10); N- acetylcysteine (NAC) group (NAC, n = 10); diabetic cardiomyopathy group (DCM, n = 10) and DCM treated with NAC group (DCM + NAC, n = 10). In DCM + NAC, rats received single intraperitoneal injection of STZ (65 mg/Kg bodyweight, dissolved in citrate buffer; Sigma- Aldrich) [22], then treated by single intraperitoneal injection of freshly prepared NAC (300 mg/Kg bodyweight) [23]; in DCM, rats were administrated with the same dosage of STZ and then received physiological saline by intraperitoneal injection (equal volume with NAC); in NAC, rats first received physiological saline (equal volume with STZ), then treated by NAC in the same time and way; In Ctrl, rats received twice intraperitoneal injection of physiological saline at corresponding time periods.

Establishment of diabetes was identified by blood glucose concentration determined by automatic analyzer (One Touch SureStep Meter, LifeScan, USA) 2 weeks after STZ injection. Blood samples were obtained from tail vein after 12-hours fasting.

### Cardiac function determination

#### ***Plasma brain natriuretic peptide (BNP) assay***

Whole blood samples were collected using EDTA-Na_2_ vacuum blood collection tubes through abdominal aorta. After centrifugation at 1500 rpm for 20 minutes, supernatant plasma was separated. Plasma BNP concentration was determined by Triage BNP Assay (Biosite) according to manufacturer’s instructions.

#### ***Hemodynamic parameters***

Rats were anesthetized by intraperitoneal injection of chloral hydrate (10%, 0.03 mL/Kg bodyweight). Invasive hemodynamic determination was conducted according to methods describe in previous study [24]. Briefly, a Mikro Tip catheter transducer (Millar Instruments) connected to Powerlab 4/25 Biological Analysis System (AD Instrument) was intubated to left ventricle through right carotid artery. Then the hemodynamic parameters including maximum rate of left ventricular pressure increase (LVdP/dt max), maximum rate of left ventricular pressure decline (LVdP/dt min), ventricular systolic pressure (LVSP) and left ventricular end- diastolic pressure (LVEDP) were measured.

#### ***Electrocardiograph (ECG) examination***

According to methods described in previous study [25], ECG examination was conducted. Rats were anesthetized by injection of 10% chloral hydrate (0.03 mL/Kg) intraperitoneally. A standard limb lead II ECG (AD instrument, Australia) was monitored and recorded by Powerlab 4/25 Biological Analysis System (AD Instrument) continuously after no external stimuli responses were found in rats. The arrhythmia vulnerability was evaluated by number of ventricular arrhythmic events (VAEs) which were presented as ventricular premature beats, ventricular tachycardia and ventricular fibrillation during 15- minutes recording [24].

### Cell culture

Neonate male Sprague–Dawley (SD) rats were supplied by the Experimental Animal Center of Xi’an Jiaotong University, China. The rats were anesthetized with chloral hydrate (10%, 0.03 mL/Kg, ip.) and sacrificed by cervical dislocation before removing the hearts. The cardiomyocytes were isolated after heart perfusion with Liberase (4.5 mg/mL, Roche), then purified and cultured as described previously [26]. Briefly, cells were placed on laminin-coated dishes and incubated for 1 hour in culture medium containing minimum essential medium (MEM) with Hank’s buffered salt solution: MEM (HyClone), 5% bovine calf serum (HyClone), 2 mM L-glutamine (Invitrogen), 1.8 mM CaCl_2_, 100 U/mL penicillin- streptomycin (Invitrogen) and 10 mM 2, 3- butanedione monoxime (Sigma- Aldrich). After that, the culture medium was replaced by fresh culture media containing MEM, 0.1 mg/mL myocyte bovine serum albumin (Sigma-Aldrich), 2 mM L-glutamin (Invitrogen) and 100 U/mL penicillin-streptomycin (Invitrogen).

### Target gene knockdown and cell treatment

#### ***Small interfering RNA (siRNA) knockdown treatment***

PERK, ATF6 and IRE1 were knocked down respectively using small interfering RNA (siRNA, Takara). Targeting sequences for each gene: PERK: 5′- CACAAACTGTATAACCGTTA-3′; AFT6: 5′-CAGCAACCAATTTATCAGTTTA-3′ and IRE1: 5′- CAGCACGGACGTCCAGTTTGA-3′ [27]. Then equal amount of siRNA was transfected into cultured cardiomyocytes(at 60% confluence) at final concentration of 12.5nM by using HiPerFect siRNA transfection reagent (Qiagen) according to the manufacturer’s instructions. The transfected cells were then incubated for 24 h prior to cell treatment.

#### ***Cell treatment***

When cell populations reached confluence at 50%-60%, the cultures were exposed to whether normal glucose (NG) concentration (5.5 mM) or high- glucose (HG) concentration (33 mM) for 48 h in accordance to previous study [28].

### Cardiomyocyte apoptosis assessment

Terminal dUTP transferase nick end labeling (TUNEL) assay was applied to detect apoptosis in cardiac tissue. Briefly, Paraffin- embedded cardiac tissue were sectioned at 5 μm, deparaffinized and then digested by proteinase K (20 μg/mL, Sigma- Aldrich, USA). After soaked in phosphate buffer saline (PBS) for 15 minutes, a TUNEL assay was performed on processed sections using TUNEL assay kit (Roche) per the manufacturer’s instructions. Apoptosis in cultured primary cardiomyocytes was assessed by flow cytometry using a Annexin V-FITC and propidiumiodide (PI) double staining. Briefly, 5 μl of Annexin V-FITC (BD) and 5 μl of PI (BD) were applied to cells suspended in binding buffer. After 15-minutes incubation at dark, a flow cytometer (FACS Calibur, BD) was used to analyze each sample.

### Intracellular ROS detection

DHE staining was utilized to detect intracellular ROS in cardiac tissue. Serial frozen sections of fresh cardiac tissue were cut to a thickness of 10 μm at -20°C. Slides were then incubated with DHE (10 μmol/L, Beyotime) at 37°C for 45 minutes in a humidified dark chamber. ROS in cultured cells were detected by DCFH-DA staining (Beyotime). DCFH-DA was diluted by DMEM to a final concentration of 10 μmol/L to incubate the cells in a 6- well plate (Corning) at 37°C for 20 minutes. Fluorescent images were captured by inverted fluorescence microscope (TE2000U, Nikon) and analyzed by software Image Pro (Version 5.0, Media Cybernetic).

### Subcellular fractionation

Method of isolation of subcellular fractions was in accordance with previous studies [29,30] but with little modifications. Cultured cardiomyocytes were washed in PBS for three times, then suspended in an isolation buffer [1 mM EDTA, 5 mM HEPES (amresco) PH = 6.8, 250 mM sucrose, protease inhibitor cocktail (amresco)], and homogenized on ice with a Dounce homogenizer (Active Motif) for 5 strokes. By spinning at 1000 g at 4°C for 6 minutes, nuclei and unbroken cells were cleared from homogenates (H1). The supernatant (S1) from H1 was further centrifuged at 8000 g for 10 minutes. The resulting supernatant (S2) was further centrifuged at 100000 g for 30 minutes to obtain pellet (P3a) (light membrane fraction) and supernatant (S3) (cytosolic fraction), which were then centrifuged at 100000 g to gain further purification. The resulting pellet (P2) (crude mitochondrial fraction) from S1 was simultaneously spun at 8000 g for 10 minutes twice to gain further purification. The resulting pellet (P3b) from P2 was then centrifuged on Percoll gradients (30% Percoll, GE) at 95000 g for 30 mintues. At last, pure mitochondria (high-density band) and mitochondria-associated endoplasmic reticulum membranes (MAMs) fraction (low-density band) were collected respectively. 20 μg proteins from each fraction layer were loaded for further western blotting analysis.

### Real- time quantitative PCR

According to manufacturer’s instructions, total RNA was extracted using RNAfast 200 Kit (Fastagen) and then reversed transcribed to cDNA using PrimeScript RT Reagent Kit (Takara). SYBR Premix Ex TaqTMII (Takara) and Prism 7500 real- time PCR detection system (Applied Biosystems) were used to perform Real time quantitative PCR. The oligonucleotide primers for target genes including GRP78, AFT4, CHOP and β-Actin [22,29] were listed in Table 1. Relative levels of mRNA were normalized to β- Actin and calculated by △cycle threshold (△Ct = CtTarget-Ctβ-actin) using Bio-Rad IQ5 software (Version 1.0, Bio-Rad).

**Table 1:** Primers for Real- time PCR

**Gene Primer**		**Sequence**	**Size**
GRP78	Forward	5′ TCAGCCCACCGTAACAAT 3′	275
	Reverse	5′ CAAACTTCTCGGCGTCAT 3′	
ATF-4	Forward	5′ CACTAGGTACCGCCAGAAGAAGA 3′	140
	Reverse	5′ AATCCGCCCTCTCTTTTAGAG 3′	
CHOP	Forward	5′ AGCTGAGTCTCTGCCTTTCG 3′	456
	Reverse	5′ TGTGGTCTC TACCTCCCTGG 3′	
β- Actin	Forward	5′ TGGCACCCAGCACAATGAA 3′	186
	Reverse	5′ CTAAGTCATAGTCCGCCTAGAAGCA 3′	

### Western blotting

According to manufacturer’s instructions, cardiac tissue or cells were homogenized in RIPA lysis buffer system (Santa Cruz) with PMSF (Santa Cruz). The supernatants were separated from homogenates after centrifugation at 12500 g, 4°C for 10 minutes. Then the sample protein concentration in supernatants was detected by BCA protein assay kit (Santa Cruz). Sample protein was subjected to 1 × SDS- PAGE loading buffer then separated by electrophoresis in SDS- polyacrylamide gel (10% or 8%), then transferred to a polyvinylidene fluoride (PVDF) membrane. Blots were then probed by specific antibodies against GRP78 (Proteintech), PERK (Cell Signaling), phosphorylated PERK (p-PERK) (Cell Signaling), ATF4 (Cell Signaling), CHOP (Proteintech), IRE1(Abcam), phosphorylated IRE1 (p-IRE1) (Abcam), ATF6 (Abcam), Sigma Receptor (Santa Cruz) and β- Actin (Santa Cruz) at 4°C overnight. After washes by Tris- buffered saline (TBS) - Tween 20 (0.02%), second antibody conjugated to HRP (Santa Cruz) was used to incubate the membranes at room temperature for 2 hours. At last, membranes were developed with Super Signal West Pico chemiluminsecence reagent (Thermo Scientific) then visualized on X-ray films. ImageJ2x (Rawak Software) was used for quantification of immunoblots.

### Statistical consideration

Results in this study were expressed as (mean ± SD) and analyzed by SPSS (version 17.0, SPSS). Significances were decided by one-way analysis of variance (ANOVA), then a LSD test was carried out. P < 0.05 was set as statistically significant.

## Results

### Diabetic animal model establishment

#### ***Fasting Blood Glucose Concentration and Bodyweight***

**Figure 1:**
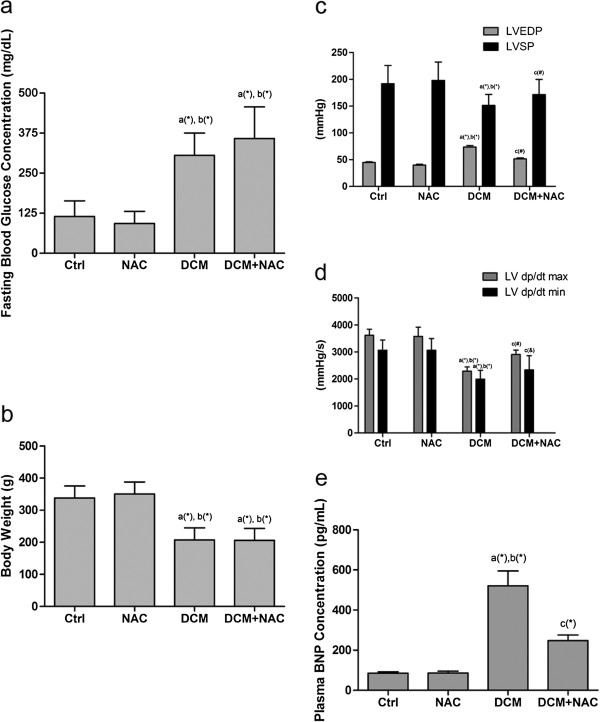
**Establishment of diabetic rat model.** Columns indicate values of Ctrl, NAC, DCM and DCM + NAC in a (mean ± SD) manner respectively. (**a**) Fasting blood glucose concentration was examined after modeling. (**b**) Body weight was measured after modeling. (**c**) and (**d**) LVESP, LVSP, LV dp/dt max and LV dp/dt min in different groups are detected by invasive hemodynamic determination. (**e**) Plasma BNP concentration (pg/mL) was determined by Triage BNP Assay. *a* Values are significantly different from Ctrl; *b* values are significantly different from NAC; *c* values are significantly different from DCM. [(&)P < 0.05; (#)P < 0.01; (*)P < 0.001].

In this study, rats with fasting plasma glucose levels higher than 300 mg/dL were deemed to be diabetic [31]. As shown in Figure 1a, fasting blood glucose levels of rats in DCM and DCM + NAC reached the diabetic rat standard of higher than 300 mg/dL; while fasting blood glucose levels of rats in Ctrl and NAC ranged from 90 to 130 mg/dL which indicated rat normal fasting blood glucose level [1]. As shown in Figure 1b, bodyweight of rats in DCM and DCM + NAC decreased significantly when compared with Ctrl and NAC.

### Impaired cardiac and electrical functions which were alleviated by NAC in diabetic rats

**Figure 2:**
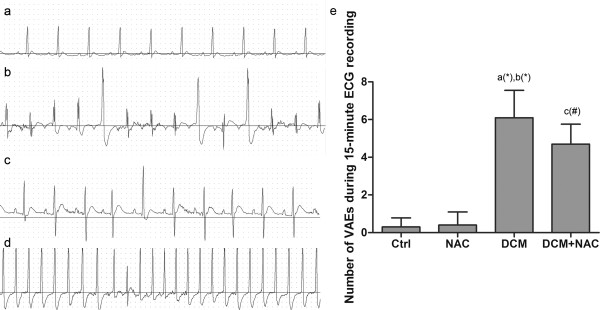
**Standard limb lead II ECG recording in different groups.** (**a**) Normal ECG. (**b**) and (**c**) premature ventricular extrasystole. (**d**) Ventricular tachycardia. (**e**) Occurring number of VAEs in Ctrl, NAC, DCM and DCM + NAC were introduced as an indicator of arrhythmia vulnerability, which were presented as (mean ± SD) in 15- minute recording and indicated by columns respectively. *a* Values are significantly different from Ctrl; *b* values are significantly different from NAC; *c* values are significantly different from DCM. [(&)P < 0.05; (#)P < 0.01; (*)P < 0.001].

Hemodynamic parameters were compared among groups. As shown in Figure 1c, 1d, and 1e, significantly increased LVEDP and plasma BNP levels but decreased LVSP, LVdP/dt max and LVdP/dt min were found in DCM compared with Ctrl and NAC. However, in DCM + NAC, NAC administration significantly reduced LVEDP and plasma BNP levels, promoted LVSP, LVdP/dt max and LVdP/dt min compared with DCM. The VAEs of rats were recorded by ECG, as demonstrated in Figure 2, significantly increased number of VAEs was observed in DCM compared with Ctrl and NAC. After NAC administration in DCM + NAC, number of VAEs decreased remarkably.

### NAC administration attenuated cardiac ROS generation in cardiac tissue from rat model of DCM

**Figure 3:**
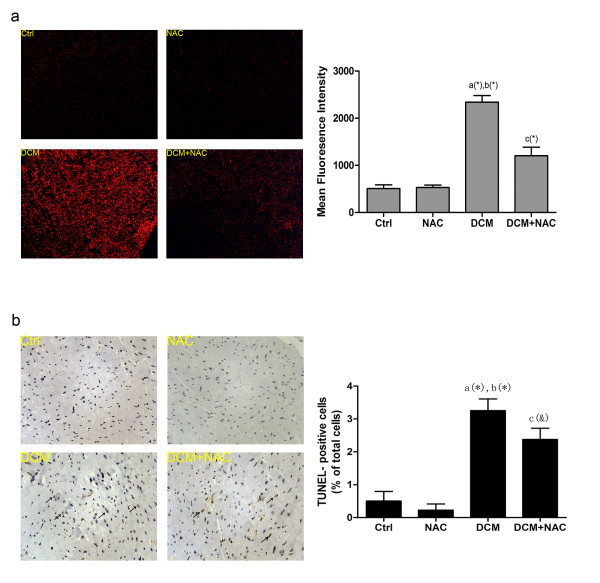
**Detection of ROS generation and cell apoptosis in cardiac tissue in different groups. **(**a**) Intracellular ROS generation detection in vivo by DHE staining. The left side shows fluorescence microscopic images of cardiac frozen sections in Ctrl, NAC, DCM and DCM + NAC. The right side demonstrates the values of fluorescence intensity which stand for level of ROS generation. (**b**) Determination of in situ apoptosis in cardiac tissue in Ctrl, NAC, DCM and DCM + NAC groups. The left side represents TUNEL assay of paraffin- embedded myocardial tissue slides in Ctrl, NAC, DCM and DCM + NAC groups. Representative photomicrographs of TUNEL staining in myocytes are indicated by black arrows. The right side shows the qualification of TUNEL- positive (yellow- brown stained) cardiomyocytes. Columns in this figure indicate detected values of Ctrl, NAC, DCM and DCM + NAC respectively in (mean ± SD) manner. *a* Values are significantly different from Ctrl; *b* values are significantly different from NAC; *c* values are significantly different from DCM. [(&)P < 0.05; (#)P < 0.01; (*)P < 0.001].

As shown in Figure 3a, ROS generation in frozen cardiac tissue sections in each group was examined in this study. DHE fluorescence staining intensity which is a specific indicator of ROS was found significantly stronger in DCM than Ctrl and NAC. As expected, DHE fluorescence staining intensity decreased significantly in DCM + NAC after intraperitoneal administration of NAC.

### ROS- mediated ER- stressinduced myocyte apoptosis in cardiac tissue from rat model of DCM

As demonstrated in Figure 3b, DCM showed a dramatically increased number of TUNEL- positive cells than Ctrl and NAC. Administration of NAC suppressed myocyte apoptosis significantly in DCM + NAC.

### Activation of ER stress and its sensors- IRE1, PERK and ATF6, in cardiac tissue from rat model of DCM

**Figure 4:**
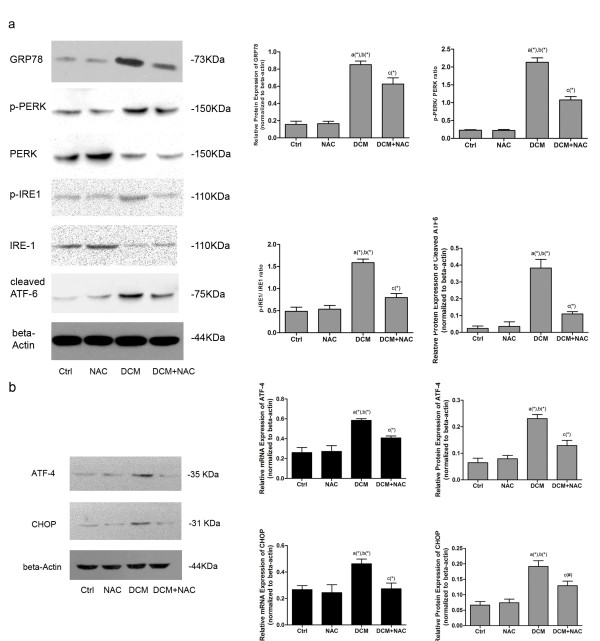
**Detection of ER stress and its sensors in cardiac tissue in different groups.** (**a**) Immunoblots of GRP78 and ER stress sensors in different groups. The left side of this figure shows immunoblots of GRP78, p-PERK, PERK, p-IRE1, IRE1, cleaved ATF6 and beta-actin from top to bottom respectively in Ctrl, NAC, DCM and DCM + NAC. The right side of this figure demonstrates quantitative analysis of immunoblots on the left side of this figure. p-PERK/PERK ratio, p-IRE1/IRE1 ratio are used to stand for activation levels of PERK and IRE1; expression levels of GRP78 and cleaved ATF6 which are normalized to beta- actin are used to stand for activation levels of GRP78 and ATF6 among different groups. (**b**) Immunoblots and real- time quantitative PCR analysis of activation of PERK branch of ER stress in different groups. The left part of this figure shows immunoblots of ATF4, CHOP and beta-actin in Ctrl, NAC, DCM and DCM + NAC respectively. The middle part of this figure shows real-time PCR analysis of mRNA expression of ATF4 and CHOP (normalized to beta- actin) in Ctrl, NAC, DCM and DCM + NAC groups respectively. The right part of this figure shows quantitative analysis of immunoblots of ATF4 and CHOP (normalized to beta- actin) in Ctrl, NAC, DCM and DCM + NAC groups respectively. Values in this figure are presented by columns in (mean ± SD) manner. *a* Values are significantly different from Ctrl; *b* values are significantly different from NAC; *c* values are significantly different from DCM. [(&)P < 0.05; (#)P < 0.01; (*)P < 0.001].

The up-regulated expression of GRP78 indicates the activation of ER stress. As for the sensors, the activation of IRE1 and PERK are in form of self- phosphorylation; ATF6 is activated in the process of cleavage. As shown in Figure 4a, GRP78 and all three ER stress sensors, IRE1, PERK and ATF6, were more dramatically activated in DCM compared with Ctrl and NAC. However, the activations were significantly inhibited by NAC administration in DCM + NAC.

### Activation of PERK signaling pathway in cardiac tissue from rat model of DCM

Compared with Ctrl and NAC, in diabetic cardiac tissue under pathological condition of hyperglycemia, the activation of PERK signaling pathway was evidenced by increased phosphorylation of PERK (Shown in Figure 4a), up- regulated expression of ATF-4 and CHOP in DCM group. NAC treatment was proposed to inhibit signal transduction in PERK signaling pathway via deactivating PERK and down- regulating expression of ATF-4 and CHOP (Figure 4b).

### Lack of PERK protected ROS- induced ER stress- mediated apoptosis more profoundly than lack of IRE1 or ATF6 in high- glucose incubated primary cadiomyocytes

**Figure 5:**
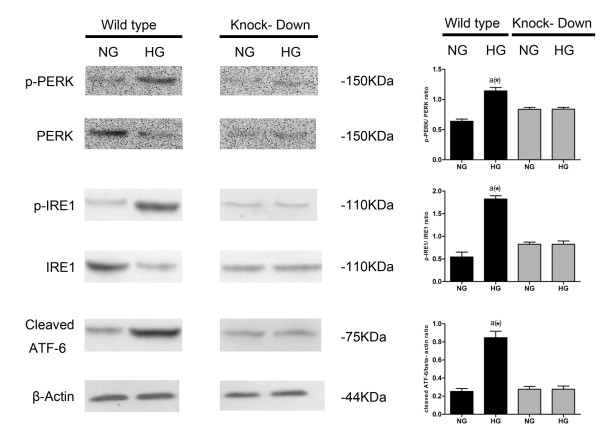
**Determination of ER stress sensors in wild type, PERK dn, IRE1 dn and ATF6 dn cardiomyocytes.** The right side of this figure demonstrates quantitative analysis of immunoblots on the left side of this figure. Columns indicate the expression level of imunoblotted proteins including p-PERK, PERK, p-IRE1, IRE1, cleaved ATF6 and beta- actin respectively. Ratio of p-PERK/PERK, p-IRE1/IRE1 and cleaved ATF6/beta- actin were used to stand for activation levels of PERK, IRE1 and ATF6 in wild type myocytes (black columns) and knocked- down myocytes (grey columns) whether incubated in normal- glucose condition or high- glucose condition. Values in this figure are presented by columns in (mean ± SD) manner. *a* Values are significantly different from NG. [(&)P < 0.05; (#)P < 0.01; (*)P < 0.001].

**Figure 6:**
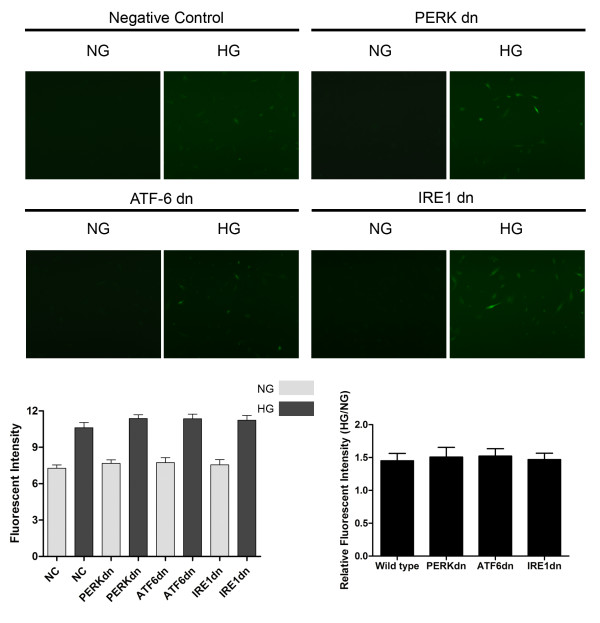
**Intracellular ROS generation detection in vitro by DCFH-DA staining.** The upper panel shows fluorescence microscopic images of cultured cardiomyocytes exposed to whether normal- glucose concentration or high- glucose concentration. As shown in the upper panel, ROS level are detected in wild type, PERK dn, ATF6 dn and IRE1 dn cardiomyocytes. The lower panel shows the fluorescent intensity (left side) and relative fluorescent intensity (right side) of DCFH-DA staining in wild type, PERK dn, ATF6 dn and IRE1 dn cardiomyocytes. Values in this figure are presented by columns in (mean ± SD) manner.

**Figure 7:**
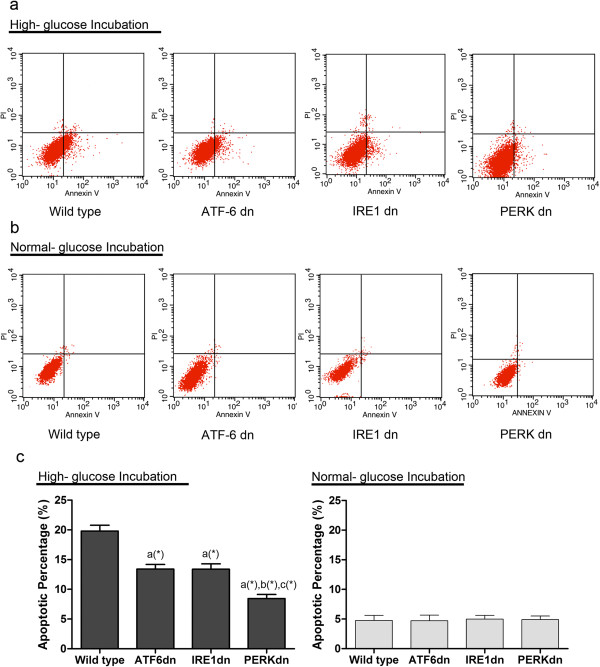
**Determination of cell apoptosis in wild type, ATF6 dn, IRE1 dn and PERK dn cardiomyocytes.** (**a**) the cell apoptosis detected by annexin V and PI double staining by flow cytometry in wild type, ATF dn, IRE1 dn and PERK dn cardiomyocytes exposed to high- glucose incubation. (**b**) the cell apoptosis detected by annexin V and PI double staining by flow cytometry in wild type, ATF dn, IRE1 dn and PERK dn cardiomyocytes exposed to normal- glucose incubation. (**c**) The columns in the lower panels show the qualification of apoptotic percentage in the charts in the upper panel. *a* Values are significantly different from Ctrl; *b* values are significantly different from NAC; *c* values are significantly different from DCM. [(&)P < 0.05; (#)P < 0.01; (*)P < 0.001].

By utilizing RNAi technique, expressions of IRE1, ATF6 and PERK were found absent respectively in IRE1dn, ATF6dn and PERKdn cardiomyocytes. There was no indication of elevated activation of these ER stress sensors (Figure 5) in responses to similar amount of high- glucose induced intracellular ROS generation (Figure 6). Furthermore, as shown in Figure 7, we found PERKdn, ATF6dn and PERKdn cardiomyocytes were all significantly more resistant to ER- stress induced cell death compared with their wild type counterparts. And interestingly, after exposure to oxidative stress, it seems that PERKdn cells were dramatically more resistant to apoptosis compared with ATF6dn or IREdn cells.

### PERK was located on MAMs rather than IRE1 or ATF6

**Figure 8:**
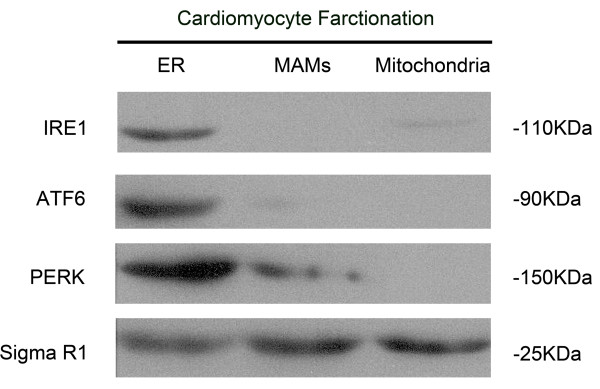
**Immunoblots of proteins obtained from subcellular fractionations of cardiomyocytes.** ER, mitochondria and MAMs fractions are isolated from whole cell lysates from cardiomyocytes. Antibodies against IRE1, ATF6 and PERK are used to detect the protein expressions of IRE1, ATF6 and PERK in cellular fractions of ER, MAMs and mitochondria. Sigma R1 is introduced as a internal reference.

Intracellular localization of ER- stress branch governors was investigated by subcellular fractionation and western blotting in cardiomyocytes. Subcellular fractions containing ER, mitochondria and MAMs were immunoblotted by antibodies against PERK, ATF6, IRE1 and Sigma Receptor respectively. As demonstrated in Figure 8, PERK was found enriched in ER and MAMs together with Sigma Receptor which is well defined as a molecular component of MAMs, also expresses in mitochondria and ER. However, the expressions of IRE1 and ATF6 were indentified only in ER rather than mitochondria and MAMs.

## Discussion

As the spread of sedentary lifestyle and obesity in the modern society, according to the data from World Health Organization (WHO), over 300 million people will suffer from diabetes mellitus by the year 2025 [32]. As one of the chronic cardiac complications, cardiovascular complications are major causes responsible for mortality of diabetes [33,34]. In diabetes- afflicted population, increased risk for cardiac dysfunction which was termed as DCM which was considered independent from other cardiovascular diseases including hypertension, congenital heart disease, valvular heart diseases and coronary artery disease [35]. Apoptosis of cardiomyocytes is considered as one of the hallmarks of DCM, taking part in pathogenesis and progression of cardiac dysfunction during DCM [5,36]. In this study, diabetes in rats was mimicked by intraperitoneal injection of STZ which selectively causes damage to islet beta cells to suppress insulin secretion. The induction of DCM was evidenced by cardiac pump and electrical dysfunctions. In the present study, in accordance with previous studies [37], DCM in diabetic rats was characterized by significant weakening of systolic and diastolic cardiac performances, associated with increased plasma BNP level which is generally used to evaluate the severity and prognosis of cardiac dysfunctions [38]. Moreover, the remarkable BNP overexpression seemed to be the best early marker for cardiac changes of DCM [39]. Rarely reported in previous studies, by electrocardiogram examination, we also found that hyperglycemia made diabetic hearts more vulnerable to arrhythmia which was evidenced by increased occurring number of VAEs. The possible mechanism was the decrease of expression ATP- sensitive potassium (K_ATP_) channels, as evidenced in both diabetic rats and high glucose treated cardiomyocytes [40], which increased the susceptibility to arrhythmia [41].

It is suggested that increased oxidative stress and cardiomyocytes apoptosis take responsibility to the pathogenesis and development of cardiovascular complications of diabetes [42]. Myocardiac apoptosis has been generally considered as a vital cause in both pathogenesis and progression of kinds of cardiac diseases such as myocardial infarction, ischemia reperfusion and DCM [37,43]. Cardiac apoptosis is also believed as a therapeutic target to improve cardiac functions by various kind of drugs [44,45]. Accumulating evidences show that ROS produced during oxidative stress results in myocytes apoptosis during DCM [46,47]. Furthermore, several studies indicated that oxidative stress is the cause of cardiomyocyte apoptosis in diabetes. By attenuating oxidative stress, previous study showed a significant decline of cardiac apoptosis in cardiac tissue in diabetic mice [48]. In our study, increased ROS generation in oxidative stress was proved as the cause of apoptotic cell death in diabetic hearts. Indeed, by DHE staining, higher intracellular ROS level was detected in cardiac tissue along with more TUNEL- positive myocytes after exposure to hyperglycemia compared with non- diabetic heart. In addition, after administration of NAC, an efficient ROS scavenger, in rats with DCM, number of TUNEL- positive myocytes decreased dramatically. These results indicated that ROS generated in excessive oxidative stress led to apoptotic events in diabetic hearts, which was in accordance with previous studies [49].

Except for two classical apoptotic signaling pathways- the death receptor and mitochondria- mediated pathways, it is believed that ER stress is another major pathway conducting cell apoptotic signals [50]. ER is an important cell organelle which performs cellular vital functions, mainly including protein synthesis, protein post- translational modification, protein folding, sorting and trafficking [51]. In responses to many stimuli, ER dysfunction would lead to accumulation of misfolded proteins which eventually results in ER stress [52]. However, when too severe, activation of ER stress would eventually lead to eukaryotic cell apoptosis [53]. GRP78, also known as immunoglobulin heavy chain binding protein (BIP), is an important molecular chaperone in recognizing and binding to unfolded proteins in ER stress [54]. Under certain pathological conditions, unfolded proteins accumulate in the ER lumen and necessitate GRP78 dissociation, then induce the UPR. After triggering ER stress, expression of GRP78 is dramatically elevated, thus, expression level of GRP78 could be used as an indicator of ER stress. In this study, the expression of GRP78 in cardiac tissue was investigated by western blotting and real- time PCR. We found that the ER stress hallmark, GPR78, was upregulated at both protein and mRNA levels in the diabetic heart when compared with normal hearts. The result showed that along with intracellular ROS generation, there was a more intensified ER stress in diabetic heart. Our findings were also in accordance with others: the up-regulated GRP78 and Bax expressions in myocytes were associated with increased apoptosis in diabetic myocardium [55]. However, after ROS blocked by NAC, the extent of ER stress was impaired and the apoptotic myocytes were then reduced, suggesting that the down- stream signaling in ER stress in DCM was mediated by upper- stream signaling from ROS, the ER stress might be the key connection between excessive ROS generation and myocyte apoptosis.

Generally, in multicellular eukaryotyes, in response to ER stress, three ER localized transmembrane signal transducers, referred as IRE1, PERK and ATF6, are activated to initiate subsequent responses [17]. Act as stress sensors, the three transducers to monitor the condition of ER. Under physiological conditions, each of these sensors is maintained inactively by binding to GRP78; however, due to ER stress, GRP78 dissociates from each transducers which triggers their activation and induction of UPR [50]. Considering the roles of IRE1, PERK and ATF6, it is possible that these receptors are fundamental to ER stress- induced apoptosis. In response to UPR or UPR derived changes in heterologous protein interactions [52], after transautophosphorylation, the activated IRE1 (p-IRE1) recruits adaptor protein- tumor necrosis factor receptor associated factor 2 (TRAF2) and apoptosis signal regulating kinase 1 (ASK1), to form IRE1-TRAF2-ASK1 complex, leading to apoptosis by activating c-Jun N-terminal kinase (JNK) and downstream mitochondria/Apaf-1- dependent caspase signaling [56,57]. In response to ER stress, PERK is activated by autophosphorylation and homomultimerization. Activated PERK (p-PERK) then phosphorylates the alpha- subunit of eIF2 (eIF2α) which then shut down translation initiation of global genes except ATF4. Under prolonged or strong ER stress, continued ATF4 expression mediates the upregulation of gene that contribute to cell apoptosis. The transcription factor C/EBP homologous protein (CHOP), whose expression strongly depends on ATF4, is primarily considered as a pro-apoptotic transcription factor [15]. The CHOP^-/-^ cells and mice exhibit decreased apoptosis in response to ER stress, indicating the significant role of this pathway [58]. By translocating the pro-apoptotic protein Bax from cytosol to mitochondria and decreasing expression of the antiapoptotic BCL-2 protein, overexpression of CHOP leads to enhanced oxidant injury and apoptosis [59,60]. Although CHOP can be activated by all three ER stress sensors, it is most strongly induced by phosphyorylation of PERK [53]. In the spontaneous diabetic rat model, remarkabely increased CHOP gene expression and phosphorylation of myocardial PERK and eIF2α were found when compared with control [61]. ATF6 is exported from ER and then translocated to Golgi apparatus under ER stress. After cleaved by Site-1 proteases (S1P) and Site-2 proteases (S2P), the cytosolic domain of ATF6 then translocates to nucleus to initiate transcription of UPR target genes [62]. In general, these genes induced on the proapoptotic phase of ER stress contribute to programmed cell death [63]. The recent study also showed that ATF6 had the ability to mediate ER stress- induced apoptosis on its own [64]. In addition, It was reported ATF6 could also induce expression of CHOP [52], the ER stress pathway involving ATF6 and CHOP played a key role in cell apoptosis under ER stress, the overexpression of ATF6 induced CHOP and following apoptosis [65]. In this study, the above- mentioned three ER stress sensors in cardiac tissue from DCM animals were investigated. We found dramatically increased p-PERK/t-PERK ratio, p-IRE1/t-IRE1 ratio and cleaved ATF6 expression, as well as the upregulated levels of the proapoptotic protein CHOP, indicating that all three ER stress sensors were activated, leading to myocyte apoptosis. However, the roles of these different ER stress sensors- mediated signaling pathway in inducing apoptosis in DCM is still unknown. This provoked our interest to propose the in vitro study by using primary cardiomyocytes.

siRNA was transfected to cardiomyocytes to knockdown PERK, IRE1 and ATF6 expression respectively. It has been documented that high glucose concentrations could increase the intracellular ROS production in myocyte, causing and promoting DCM [66,67]. In this study, as shown in Figure 6, we also found that high glucose concentration (33 mM) caused an increased generation of ROS production in both cultured wild type and the three ER sensors knockdown cardiomyocytes. There was no significant difference in ROS levels between wild type, PERKdn, IRE1dn and ATF6dn cardiomyocytes under high glucose conditions. However, compared with their wild type counterparts, all PERKdn, IRE1dn and ATF6dn cardiomyocytes exhibited the capacity of resistance to apoptosis under the similar stimuli of ROS generated by high- glucose incubation. Interestingly, under similar stimuli of ROS, PERK deficiency showed stronger protection against cell death compared with IRE1 or ATF6 deficiency. This result suggested that ROS- induced ER- stress mediated myocytes apoptosis relied more predominantly on PERK rather than IRE1 or ATF6 governed signaling pathway. However, the possible mechanism of this phenomenon is still obscure.

Under pathological condition of diabetes, increased glucose is processed by mitochondrial glucose oxidation, as a byproduct of electron transport, excessive ROS was generated [68]. The link between mitochondria and PERK signaling may reveal the above- mentioned phenomenon that the stronger protection effect of PERK deficiency. MAMs is the functional and physical contact site between ER and mitochondria. The function of MAMs includes calcium signaling, lipid metabolism, inflammation and so on [69]. Recent studies suggested that there was a group of MAMs proteins which were ER protein folding chaperones and redox regulators such as Sigma Receptor and calnexin [70]. In addition, considering PERK acts as a transcriptional regulator in redox homeostasis, we speculated that PERK located on MAMs rather than IRE1 or ATF6. Subcellular fractionation was used to isolate ER, mitochondria and MAMs from cardiomyocytes. Results from western blotting confirmed our speculation that PERK was enriched in ER and MAMs, but IRE1 and ATF6 were found only located in ER rather than MAMs. MAMs’ crucial role of conducting apoptosis signals was demonstrated by previous study: after ER- mitochondria contact points were bound and blocked by keratin- binding protein, cells were resistance to apoptosis after oxidative stress exposure [71].

**Figure 9:**
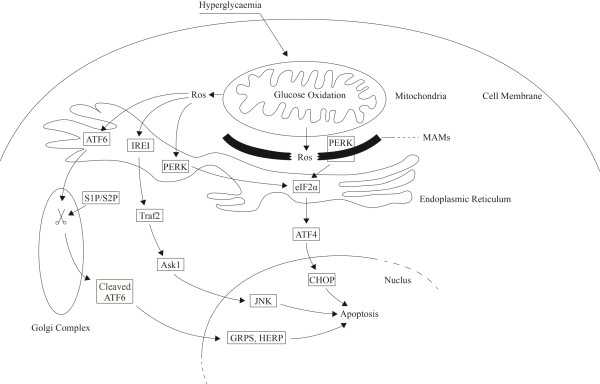
**Schematic diagram demonstrating speculated roles of ER stress signaling pathways in diabetic cardiomyopathy.** Firstly, under pathological condition of hyperglycemia in myocytes, ROS is generated by glucose oxidation in mitochondria. Then ROS is released to ER and sensed by three independent ER stress sensors- ATF6, IER1 and PERK to induce ER stress. Under excessive ER stress, apoptotic signals are transducted by ATF6, IRE1 and PERK signaling pathways to induce myocytes apoptosis. Meanwhile, as a component of MAMs proteins, PERK also receives signals from ROS affecting MAMs. Thus, as PERK signaling pathway received double stimulation of ROS in ER and MAMs, PERK is proposed the major signaling pathway transducting apoptotic signals in ROS- mediated ER stress- induced cell apoptosis in DCM. (Non-specific or cross- talk signaling transduction by ER stress sensors is not shown in this figure).

Thus, as demonstrated in Figure 9, it is relatively clear for us to speculate the notion that PERK may govern the major signaling pathway in inducing apoptosis in DCM. The most likely mechanisms are shown as follows. Hyperglycemia causes abnormal glucose metabolism in cardiomyocytes. After mitochondrial glucose oxidation, ROS is produced and released to disturb the redox balance and result in oxidative stress in myocytes. After interaction with proteins in ER lumen, as misfolded protein accumulating, ER stress signaling is initiated. Three ER stress sensors, PERK, IRE1 and ATF6 are then activated in response to ROS to conduct apoptotic signals to eliminate irrevocable damaged myocytes through separate and particular pathways. In this process, as a component of ER and a component of MAMs, PERK may play a important role in linking the ROS generation and ER stress induced apoptotic events.

ER stress is considered playing an important role in inducing myocytes apoptosis in DCM. This study showed that deletion of PERK exhibited stronger protective effect against HG-induced cell death than deletion of other two arms, namely ATF6 and IRE1, due to its localization and enrichment on MAMs. However, there were also several limitations which could be improved in the further studies. Utility of ER sensors knock-out animal would be helpful in supporting and proving the current conclusions. Other studies should also be conducted to specifically address how PERK is located at this ER-mitochondria junction and what function it can carry out. And more interestingly, whether and how the arrhythmia is correlated with sarcoplasmic reticulum (SR) calcium mishandling, another important aspect of ER stress, has become a novel and potential research topic for our group in the very near future.

## Conclusions

This study suggests a new recondition of signaling transduction in explaining mechanism of DCM:

1. ROS- induced ER stress mediated myocyte apoptosis may play an important role in DCM which is characterized by cardiac pump and electrical dysfunctions.

2. All three ER stress sensors, IRE1, ATF6 and PERK, governed signaling pathway participate in ER stress- induced cell apoptosis in DCM.

3. Rather than IRE1 or ATF6, PERK governed signaling pathway is probably the major pathway conducting apoptotic signaling in ROS- induced ER stress mediated myocyte apoptosis in DCM.

## Abbreviations

ER: Endoplasmic reticulum; ROS: Reactive oxygen species; DCM: Diabetic cardiomyopathy; NAC: N- acetylcysteine; STZ: Streptozocin; BNP: Plasma brain natriuretic peptide; NG: Normal glucose; HG: High glucose; MAMs: Mitochondria-associated endoplasmic reticulum membranes; PERK: Protein kinase RNA (PKR)- like ER kinase; IRE1: Inositol- requiring enzyme-1; ATF6: Activating transcription factor-6; ATF4: Activating transcription factor-4; GRP78: Glucose-related protein 78; eIF2: Eukaryotic initiation factor 2; CHOP: C/EBP homologous protein; Bax: BCL-2 associated X protein; BCL-2: B cell CLL/lymphoma; SR: Sarcoplasmic reticulum.

## Competing interests

The authors declare that they have no competing interests.

## Authors’ contributions

J-HX is the guarantor of this work, and as such, she had full access to all the data in this study and takes full responsibility for the integrity of the data and accuracy of the data analysis. Z-WL and H-TZ researched data and wrote the manuscript; K-LC researched data; CQ reviewed and edited the manuscript; JW reviewed the manuscript; XD reviewed the manuscript. All authors read and approved the final manuscript.

## References

[B1] Boudina S Abel ED Diabetic cardiomyopathy revisited Circulation 2007 115 25 3213 3223 17592090 10.1161/CIRCULATIONAHA.106.679597

[B2] Boudina S Abel ED Diabetic cardiomyopathy, causes and effects Rev Endocr Metab Disord 2010 11 1 31 39 20180026 10.1007/s11154-010-9131-7 PMC2914514

[B3] Bertoni AG Tsai A Kasper EK Brancati FL Diabetes and idiopathic cardiomyopathy: a nationwide case–control study Diabetes Care 2003 26 10 2791 2795 14514581 10.2337/diacare.26.10.2791

[B4] Nichols GA Hillier TA Erbey JR Brown JB Congestive heart failure in type 2 diabetes: prevalence, incidence, and risk factors Diabetes Care 2001 24 9 1614 1619 11522708 10.2337/diacare.24.9.1614

[B5] Cai L Kang YJ Cell death and diabetic cardiomyopathy Cardiovasc Toxicol 2003 3 3 219 228 14555788 10.1385/ct:3:3:219

[B6] Engel D Peshock R Armstong RC Sivasubramanian N Mann DL Cardiac myocyte apoptosis provokes adverse cardiac remodeling in transgenic mice with targeted TNF overexpression Am J Physiol Heart Circ Physiol 2004 287 3 H1303 H1311 15317679 10.1152/ajpheart.00053.2004

[B7] Li Z Zhang T Dai H Liu G Wang H Sun Y Zhang Y Ge Z Involvement of endoplasmic reticulum stress in myocardial apoptosis of streptozocin-induced diabetic rats J Clin Biochem Nutr 2007 41 1 58 67 18392099 10.3164/jcbn.2007008 PMC2274987

[B8] Barouch LA Berkowitz DE Harrison RW O’Donnell CP Hare JM Disruption of leptin signaling contributes to cardiac hypertrophy independently of body weight in mice Circulation 2003 108 6 754 759 12885755 10.1161/01.CIR.0000083716.82622.FD

[B9] Wold LE Ren J Streptozotocin directly impairs cardiac contractile function in isolated ventricular myocytes via a p38 map kinase-dependent oxidative stress mechanism Biochem Biophys Res Commun 2004 318 4 1066 1071 15147982 10.1016/j.bbrc.2004.04.138

[B10] Cai L Suppression of nitrative damage by metallothionein in diabetic heart contributes to the prevention of cardiomyopathy Free Radic Biol Med 2006 41 6 851 861 16934665 10.1016/j.freeradbiomed.2006.06.007

[B11] Saraiva RM Minhas KM Zheng M Pitz E Treuer A Gonzalez D Schuleri KH Vandegaer KM Barouch LA Hare JM Reduced neuronal nitric oxide synthase expression contributes to cardiac oxidative stress and nitroso-redox imbalance in ob/ob mice Nitric Oxide : Biology and Chemistry / Official Journal of the Nitric Oxide Society 2007 16 3 331 338 17307368 10.1016/j.niox.2006.12.001 PMC2754218

[B12] Kwon SH Pimentel DR Remondino A Sawyer DB Colucci WS H(2)O(2) regulates cardiac myocyte phenotype via concentration-dependent activation of distinct kinase pathways J Mol Cell Cardiol 2003 35 6 615 621 12788379 10.1016/s0022-2828(03)00084-1

[B13] Brownlee M The pathobiology of diabetic complications: a unifying mechanism Diabetes 2005 54 6 1615 1625 15919781 10.2337/diabetes.54.6.1615

[B14] Malhotra JD Kaufman RJ The endoplasmic reticulum and the unfolded protein response Semin Cell Dev Biol 2007 18 6 716 731 18023214 10.1016/j.semcdb.2007.09.003 PMC2706143

[B15] Xu J Zhou Q Xu W Cai L Endoplasmic reticulum stress and diabetic cardiomyopathy Exp Diabetes Res 2012 2012 827971 22144992 10.1155/2012/827971 PMC3226330

[B16] Nakagawa T Zhu H Morishima N Li E Xu J Yankner BA Yuan J Caspase-12 mediates endoplasmic-reticulum-specific apoptosis and cytotoxicity by amyloid-beta Nature 2000 403 6765 98 103 10638761 10.1038/47513

[B17] Malhotra JD Kaufman RJ Endoplasmic reticulum stress and oxidative stress: a vicious cycle or a double-edged sword? Antioxid Redox Signal 2007 9 12 2277 2293 17979528 10.1089/ars.2007.1782

[B18] Qi XF Zheng L Lee KJ Kim DH Kim CS Cai DQ Wu Z Qin JW Yu YH Kim SK HMG-CoA reductase inhibitors induce apoptosis of lymphoma cells by promoting ROS generation and regulating Akt, Erk and p38 signals via suppression of mevalonate pathway Cell Death & Disease 2013 4 e518 23449454 10.1038/cddis.2013.44 PMC3734846

[B19] Ding W Yang L Zhang M Gu Y Reactive oxygen species-mediated endoplasmic reticulum stress contributes to aldosterone-induced apoptosis in tubular epithelial cells Biochem Biophys Res Commun 2012 418 3 451 456 22281495 10.1016/j.bbrc.2012.01.037

[B20] Higa A Chevet E Redox signaling loops in the unfolded protein response Cell Signal 2012 24 8 1548 1555 22481091 10.1016/j.cellsig.2012.03.011

[B21] Senkal CE Ponnusamy S Bielawski J Hannun YA Ogretmen B Antiapoptotic roles of ceramide-synthase-6-generated C16-ceramide via selective regulation of the ATF6/CHOP arm of ER-stress-response pathways FASEB Journal : Official Publication of the Federation of American Societies for Experimental Biology 2010 24 1 296 308 19723703 10.1096/fj.09-135087 PMC2797032

[B22] Li Z Zhang T Dai H Liu G Wang H Sun Y Zhang Y Ge Z Endoplasmic reticulum stress is involved in myocardial apoptosis of streptozocin-induced diabetic rats J Endocrinol 2008 196 3 565 572 18310452 10.1677/JOE-07-0230

[B23] Downs I Liu J Aw TY Adegboyega PA Ajuebor MN The ROS scavenger, NAC, regulates hepatic Valpha14iNKT cells signaling during Fas mAb-dependent fulminant liver failure PLoS One 2012 7 6 e38051 22701598 10.1371/journal.pone.0038051 PMC3368940

[B24] Bocchi L Savi M Graiani G Rossi S Agnetti A Stillitano F Lagrasta C Baruffi S Berni R Frati C Growth factor-induced mobilization of cardiac progenitor cells reduces the risk of arrhythmias, in a rat model of chronic myocardial infarction PLoS One 2011 6 3 e17750 21445273 10.1371/journal.pone.0017750 PMC3060871

[B25] Soraya H Khorrami A Garjani A Maleki-Dizaji N Acute treatment with metformin improves cardiac function following isoproterenol induced myocardial infarction in rats Pharmacological Reports : PR 2012 64 6 1476 1484 23406758 10.1016/s1734-1140(12)70945-3

[B26] Zuo L Youtz DJ Wold LE Particulate matter exposure exacerbates high glucose-induced cardiomyocyte dysfunction through ROS generation PLoS One 2011 6 8 e23116 21850256 10.1371/journal.pone.0023116 PMC3151271

[B27] Jiang Y Zhang Y Wark L Ortiz E Lim S He H Wang W Medeiros D Lin D Wolfberry Water Soluble Phytochemicals Down-Regulate ER Stress Biomarkers and Modulate Multiple Signaling Pathways Leading To Inhibition of Proliferation and Induction of Apoptosis in Jurkat Cells Journal of Nutrition & Food Sciences 2011 S2 PMC3368523 22685690

[B28] Kumar S Kain V Sitasawad SL High glucose-induced Ca2+ overload and oxidative stress contribute to apoptosis of cardiac cells through mitochondrial dependent and independent pathways Biochimica Biophysica Acta 2012 1820 7 907 920 10.1016/j.bbagen.2012.02.010 22402252

[B29] Verfaillie T Rubio N Garg AD Bultynck G Rizzuto R Decuypere JP Piette J Linehan C Gupta S Samali A PERK is required at the ER-mitochondrial contact sites to convey apoptosis after ROS-based ER stress Cell Death Differ 2012 19 11 1880 1891 22705852 10.1038/cdd.2012.74 PMC3469056

[B30] Griffin S Clarke D McCormick C Rowlands D Harris M Signal peptide cleavage and internal targeting signals direct the hepatitis C virus p7 protein to distinct intracellular membranes J Virol 2005 79 24 15525 15536 16306623 10.1128/JVI.79.24.15525-15536.2005 PMC1315988

[B31] Li Y Zhang Y Liu DB Liu HY Hou WG Dong YS Curcumin attenuates diabetic neuropathic pain by downregulating TNF-alpha in a rat model Int J Med Sci 2013 10 4 377 381 23471081 10.7150/ijms.5224 PMC3590595

[B32] King H Aubert RE Herman WH Global burden of diabetes, 1995–2025: prevalence, numerical estimates, and projections Diabetes Care 1998 21 9 1414 1431 9727886 10.2337/diacare.21.9.1414

[B33] Papa G Degano C Iurato MP Licciardello C Maiorana R Finocchiaro C Macrovascular complication phenotypes in type 2 diabetic patients Cardiovasc Diabetol 2013 12 20 23331854 10.1186/1475-2840-12-20 PMC3558439

[B34] Coccheri S Approaches to prevention of cardiovascular complications and events in diabetes mellitus Drugs 2007 67 7 997 1026 17488145 10.2165/00003495-200767070-00005

[B35] Fang ZY Prins JB Marwick TH Diabetic cardiomyopathy: evidence, mechanisms, and therapeutic implications Endocr Rev 2004 25 4 543 567 15294881 10.1210/er.2003-0012

[B36] Baraka A AbdelGawad H Targeting apoptosis in the heart of streptozotocin-induced diabetic rats J Cardiovasc Pharmacol Ther 2010 15 2 175 181 20133494 10.1177/1074248409356557

[B37] Yu W Wu J Cai F Xiang J Zha W Fan D Guo S Ming Z Liu C Curcumin alleviates diabetic cardiomyopathy in experimental diabetic rats PLoS One 2012 7 12 e52013 23251674 10.1371/journal.pone.0052013 PMC3522633

[B38] Gaggin HK Januzzi JL Jr Biomarkers and diagnostics in heart failure Biochim Biophys Acta 2013 10.1016/j.bbadis.2012.12.014 23313577

[B39] Nunes S Soares E Fernandes J Viana S Carvalho E Pereira FC Reis F Early cardiac changes in a rat model of prediabetes: brain natriuretic peptide overexpression seems to be the best marker Cardiovasc Diabetol 2013 12 44 23497124 10.1186/1475-2840-12-44 PMC3599663

[B40] Chen ZC Cheng YZ Chen LJ Cheng KC Li Y Cheng J Increase of ATP-sensitive potassium (K(ATP)) channels in the heart of type-1 diabetic rats Cardiovasc Diabetol 2012 11 8 22257425 10.1186/1475-2840-11-8 PMC3274424

[B41] Smith S Curran J Hund TJ Mohler PJ Defects in cytoskeletal signaling pathways, arrhythmia, and sudden cardiac death Front Physiol 2012 3 122 22586405 10.3389/fphys.2012.00122 PMC3343379

[B42] Cai L Kang YJ Oxidative stress and diabetic cardiomyopathy: a brief review Cardiovasc Toxicol 2001 1 3 181 193 12213971 10.1385/ct:1:3:181

[B43] Neuss M Crow MT Chesley A Lakatta EG Apoptosis in cardiac disease–what is it–how does it occur Cardiovascular Drugs and Therapy / Sponsored by the International Society of Cardiovascular Pharmacotherapy 2001 15 6 507 523 11916360 10.1023/a:1013715704835

[B44] Jie B Zhang X Wu X Xin Y Liu Y Guo Y Neuregulin-1 suppresses cardiomyocyte apoptosis by activating PI3K/Akt and inhibiting mitochondrial permeability transition pore Mol Cell Biochem 2012 370 1–2 35 43 22886427 10.1007/s11010-012-1395-7

[B45] Zhang Z Yu B Tao GZ Apelin protects against cardiomyocyte apoptosis induced by glucose deprivation Chin Med J (Engl) 2009 122 19 2360 2365 20079140

[B46] Ferreira FM Palmeira CM Seica R Moreno AJ Santos MS Diabetes and mitochondrial bioenergetics: alterations with age J Biochem Mol Toxicol 2003 17 4 214 222 12898645 10.1002/jbt.10081

[B47] Ghosh S Pulinilkunnil T Yuen G Kewalramani G An D Qi D Abrahani A Rodrigues B Cardiomyocyte apoptosis induced by short-term diabetes requires mitochondrial GSH depletion Am J Physiol Heart Circ Physiol 2005 289 2 H768 776 15805231 10.1152/ajpheart.00038.2005

[B48] Li CJ Zhang QM Li MZ Zhang JY Yu P Yu DM Attenuation of myocardial apoptosis by alpha-lipoic acid through suppression of mitochondrial oxidative stress to reduce diabetic cardiomyopathy Chin Med J (Engl) 2009 122 21 2580 2586 19951573

[B49] Fiordaliso F Bianchi R Staszewsky L Cuccovillo I Doni M Laragione T Salio M Savino C Melucci S Santangelo F Antioxidant treatment attenuates hyperglycemia-induced cardiomyocyte death in rats J Mol Cell Cardiol 2004 37 5 959 968 15522273 10.1016/j.yjmcc.2004.07.008

[B50] Szegezdi E Logue SE Gorman AM Samali A Mediators of endoplasmic reticulum stress-induced apoptosis EMBO Rep 2006 7 9 880 885 16953201 10.1038/sj.embor.7400779 PMC1559676

[B51] Moserova I Kralova J Role of ER stress response in photodynamic therapy: ROS generated in different subcellular compartments trigger diverse cell death pathways PLoS One 2012 7 3 e32972 22403731 10.1371/journal.pone.0032972 PMC3293927

[B52] Ron D Walter P Signal integration in the endoplasmic reticulum unfolded protein response Nat Rev Mol Cell Biol 2007 8 7 519 529 17565364 10.1038/nrm2199

[B53] Scull CM Tabas I Mechanisms of ER stress-induced apoptosis in atherosclerosis Arterioscler Thromb Vasc Biol 2011 31 12 2792 2797 22096099 10.1161/ATVBAHA.111.224881 PMC3220876

[B54] Lee AS The ER chaperone and signaling regulator GRP78/BiP as a monitor of endoplasmic reticulum stress Methods (San Diego, Calif) 2005 35 4 373 381 10.1016/j.ymeth.2004.10.010 15804610

[B55] Shi FH Cheng YS Dai DZ Peng HJ Cong XD Dai Y Depressed calcium-handling proteins due to endoplasmic reticulum stress and apoptosis in the diabetic heart are attenuated by argirein Naunyn Schmiedebergs Arch Pharmacol 2013 386 6 521 531 23525487 10.1007/s00210-013-0852-5

[B56] Zheng QY Li PP Jin FS Yao C Zhang GH Zang T Ai X Ursolic acid induces ER stress response to activate ASK1-JNK signaling and induce apoptosis in human bladder cancer T24 cells Cell Signal 2013 25 1 206 213 23000344 10.1016/j.cellsig.2012.09.012

[B57] Nishitoh H Matsuzawa A Tobiume K Saegusa K Takeda K Inoue K Hori S Kakizuka A Ichijo H ASK1 is essential for endoplasmic reticulum stress-induced neuronal cell death triggered by expanded polyglutamine repeats Genes Dev 2002 16 11 1345 1355 12050113 10.1101/gad.992302 PMC186318

[B58] Zinszner H Kuroda M Wang X Batchvarova N Lightfoot RT Remotti H Stevens JL Ron D CHOP is implicated in programmed cell death in response to impaired function of the endoplasmic reticulum Genes Dev 1998 12 7 982 995 9531536 10.1101/gad.12.7.982 PMC316680

[B59] Oyadomari S Mori M Roles of CHOP/GADD153 in endoplasmic reticulum stress Cell Death Differ 2004 11 4 381 389 14685163 10.1038/sj.cdd.4401373

[B60] Gotoh T Terada K Oyadomari S Mori M hsp70-DnaJ chaperone pair prevents nitric oxide- and CHOP-induced apoptosis by inhibiting translocation of Bax to mitochondria Cell Death Differ 2004 11 4 390 402 14752510 10.1038/sj.cdd.4401369

[B61] Lakshmanan AP Harima M Suzuki K Soetikno V Nagata M Nakamura T Takahashi T Sone H Kawachi H Watanabe K The hyperglycemia stimulated myocardial endoplasmic reticulum (ER) stress contributes to diabetic cardiomyopathy in the transgenic non-obese type 2 diabetic rats: a differential role of unfolded protein response (UPR) signaling proteins Int J Biochem Cell Biol 2013 45 2 438 447 23032698 10.1016/j.biocel.2012.09.017

[B62] Ye J Rawson RB Komuro R Chen X Dave UP Prywes R Brown MS Goldstein JL ER stress induces cleavage of membrane-bound ATF6 by the same proteases that process SREBPs Mol Cell 2000 6 6 1355 1364 11163209 10.1016/s1097-2765(00)00133-7

[B63] Glembotski CC Endoplasmic reticulum stress in the heart Circ Res 2007 101 10 975 984 17991891 10.1161/CIRCRESAHA.107.161273

[B64] Morishima N Nakanishi K Nakano A Activating transcription factor-6 (ATF6) mediates apoptosis with reduction of myeloid cell leukemia sequence 1 (Mcl-1) protein via induction of WW domain binding protein 1 J Biol Chem 2011 286 40 35227 35235 21841196 10.1074/jbc.M111.233502 PMC3186435

[B65] Gotoh T Oyadomari S Mori K Mori M Nitric oxide-induced apoptosis in RAW 264.7 macrophages is mediated by endoplasmic reticulum stress pathway involving ATF6 and CHOP J Biol Chem 2002 277 14 12343 12350 11805088 10.1074/jbc.M107988200

[B66] Cai L Wang Y Zhou G Chen T Song Y Li X Kang YJ Attenuation by metallothionein of early cardiac cell death via suppression of mitochondrial oxidative stress results in a prevention of diabetic cardiomyopathy J Am Coll Cardiol 2006 48 8 1688 1697 17045908 10.1016/j.jacc.2006.07.022

[B67] Ku PM Chen LJ Liang JR Cheng KC Li YX Cheng JT Molecular role of GATA binding protein 4 (GATA-4) in hyperglycemia-induced reduction of cardiac contractility Cardiovasc Diabetol 2011 10 57 21702924 10.1186/1475-2840-10-57 PMC3141394

[B68] Wu M Yang S Elliott MH Fu D Wilson K Zhang J Du M Chen J Lyons T Oxidative and endoplasmic reticulum stresses mediate apoptosis induced by modified LDL in human retinal Muller cells Invest Ophthalmol Vis Sci 2012 53 8 4595 4604 22678501 10.1167/iovs.12-9910 PMC3394695

[B69] Raturi A Simmen T Where the endoplasmic reticulum and the mitochondrion tie the knot: the mitochondria-associated membrane (MAM) Biochim Biophys Acta 2013 1833 1 213 224 22575682 10.1016/j.bbamcr.2012.04.013

[B70] Hayashi T Rizzuto R Hajnoczky G Su TP MAM: more than just a housekeeper Trends Cell Biol 2009 19 2 81 88 19144519 10.1016/j.tcb.2008.12.002 PMC2750097

[B71] Cerqua C Anesti V Pyakurel A Liu D Naon D Wiche G Baffa R Dimmer KS Scorrano L Trichoplein/mitostatin regulates endoplasmic reticulum-mitochondria juxtaposition EMBO Rep 2010 11 11 854 860 20930847 10.1038/embor.2010.151 PMC2966954

